# Delineation of the cognitive and neuropsychiatric features of Pisa syndrome in dementia with Lewy bodies

**DOI:** 10.3389/fnagi.2025.1637930

**Published:** 2025-11-19

**Authors:** Zhou Su, Jun Kuai, Tingting Yi, Hao Wu, Jinghuan Gan, Zhihong Shi, Shuai Liu, Yong Ji

**Affiliations:** 1 Department of Neurology, The First Affiliated Hospital of Xinxiang Medical University, Xinxiang, Henan, China; 2 Department of Gastroenterology, The First Affiliated Hospital of Xinxiang Medical University, Xinxiang, Henan, China; 3 Tianjin Key Laboratory of Cerebrovascular and Neurodegenerative Diseases, Department of Neurology, Tianjin Huanhu Hospital, Tianjin Dementia Institute, Tianjin, China; 4 Department of Neurology, Beijing Friendship Hospital, Capital Medical University, Beijing, China

**Keywords:** Pisa syndrome, dementia with Lewy bodies, cognitive impairment, neuropsychiatric symptoms, caregiver burden

## Abstract

**Background:**

Pisa syndrome (PS), characterized by trunk lateral flexion, remains rarely reported in dementia with Lewy bodies (DLB). While its pathogenesis is multifactorial, underlying mechanisms are not fully understood. Given the established link between postural control and cognition in aging populations, cognitive impairment has been implicated in PS development. Notably, no studies have investigated the potential contributions of cognitive dysfunction and neuropsychiatric symptoms to the development of PS in DLB patients or its relationship with caregiver burden.

**Methods:**

This study included 35 DLB patients with PS and 183 DLB patients without PS. We compared cognitive function across different domains using the Montreal Cognitive Assessment (MoCA) and its subdomains, and the Clock Drawing Test (CDT), and dementia severity using the Clinical Dementia Rating (CDR). The prevalence and severity of neuropsychiatric symptoms and caregiver distress were measured using the Neuropsychiatric Inventory (NPI) and Zarit Burden Interview (ZBI).

**Results:**

The patients in the PS group showed significantly worse performances in attention (*p* = 0.012), visuospatial/executive abilities (*p* = 0.013), and lower scores on the CDT (*p* = 0.007). The PS group demonstrated significantly elevated NPI total scores (*p* = 0.008), with higher frequency and severity of delusions (*p* = 0.006 and *p* = 0.008), hallucinations (*p* = 0.004 and *p* < 0.001), and aberrant motor behaviors (*p* = 0.020 and *p* = 0.006). The PS group also had significantly higher ZBI scores (*p* = 0.023). Caregivers in the PS group reported greater distress and burden related to delusion (*p* = 0.030), hallucination (*p* < 0.001), anxiety (*p* = 0.020), aberrant motor behavior (*p* = 0.001), and sleep disturbance (*p* = 0.009).

**Conclusion:**

Our study reveals that PS in DLB is associated with specific deficits in attention and visuospatial/executive function, alongside more severe neuropsychiatric symptoms. These findings highlight the need for comprehensive management targeting both postural control and neuropsychiatric issues to alleviate caregiver burden. Future longitudinal studies are warranted to clarify the causal nature of these relationships.

## 1 Introduction

Pisa syndrome (PS) is a rare postural deformity characterized by persistent lateral trunk flexion, appearing or worsening while sitting, standing, or walking, and can be improved or completely mitigated by supine positioning and passive mobilization (Doherty et al., 2011). PS was first used to describe a secondary trunk muscle tone disorder after treatment with antipsychotic drugs (Ekbom et al., 1972). Subsequently, PS was widely used for patients in Alzheimer’s disease (Woo et al., 2020), Parkinson’s disease (PD) (Castrioto et al., 2014), secondary parkinsonism (Marsili et al., 2019; Su et al., 2022), and other neurological disorders (Marchione et al., 2014; Pandey, 2014).

Postural control relies on the interactive network involving the cortex, basal ganglia, and brainstem, integrating motor (trunk muscles), sensory (visual, vestibular, and proprioceptive), and cognitive functions (Barone et al., 2016; Tinazzi et al., 2016). The development of PS is multifactorial, involving potential central and peripheral mechanisms that vary across different underlying diseases (Doherty et al., 2011). Current evidences (Tinazzi et al., 2016) suggest that dysfunction of the nigrostriatal pathway, impaired sensory information processing, cognitive deficits, and altered somatosensory orientation perception contribute to the pathophysiology of PS.

Studies have demonstrated that PS is significantly associated with alterations in executive function, attention, language, visuospatial perception, and visuospatial processing in PD patients (Amboni et al., 2013; Vitale et al., 2016; Artusi et al., 2019; Liu et al., 2019a). Cognitive impairment, a common clinical feature in both dementia with Lewy bodies (DLB) and Parkinson’s disease dementia (PDD), exhibits overlapping affected cognitive domains (Walker et al., 2019). However, compared to PDD, DLB patients present more pronounced deficits in attention, executive function, visuospatial processing, language, and nonverbal memory, with visuospatial and perceptual impairments often manifesting at earlier stages (Smirnov et al., 2020). These findings highlight the need to explore the potential relationship between domain-specific cognitive dysfunction and postural abnormalities in DLB. Additionally, neuropsychiatric symptoms (NPS) differ between PD patients with and without PS (Vitale et al., 2016). Research by Tinazzi et al. (2015) suggests that NPS may influence PS development in PD. The Diagnostic and Treatment Guidelines for DLB recognize diverse NPS as supportive clinical features, which are recognized high-risk factors for dementia progression and may precede overt cognitive decline (McKeith et al., 2017; Guo et al., 2023; Kang et al., 2023).

Beyond the motor impairment, PS profoundly impacts patients’ daily functioning and quality of life (QoL) (Tinazzi et al., 2016). The postural deformity can lead to difficulties with balance and gait, significantly increasing the risk of falls and consequent injuries (Tinazzi et al., 2016; Gonzalez et al., 2023). It can also cause chronic back pain and reduce mobility, further limiting patients’ independence in activities of daily living. This increased dependency, coupled with the visible deformity, contributes to social stigma and isolation. Consequently, the management of PS places a substantial additional burden on caregivers, who must provide physical assistance, ensure safety to prevent falls, and cope with the psychological distress of their care recipients.

Building on prior evidence, this cross-sectional study aims to compare cognitive profiles and neuropsychiatric symptom variations between DLB patients with and without PS. The findings may provide clinical insights for improving postural stability, preventing falls, enhancing daily living capacity and QoL, and reducing caregiver burden.

## 2 Materials and methods

### 2.1 Study population

This multicenter clinical cross-sectional study involved data collected from neurological dementia clinics and inpatient wards between May 2012 and October 2024. Participants were categorized into two groups based on the presence or absence of PS: DLB patients with PS (*n* = 35) and DLB patients without PS (*n* = 183). This study was approved by the Committee for Medical Research Ethics at Tianjin Huanhu Hospital (ID: 2023–158). Informed consent was obtained from all participants or their legal guardians in accordance with the ethical principles of the Helsinki Declaration.

### 2.2 Enrollment and exclusion criteria for DLB patients

DLB patients were included if they met the following criteria: (1) DLB patients were diagnosed according to the fourth International Consensus Criteria for the diagnosis and management of dementia with Lewy bodies (McKeith et al., 2017). (2) Patients presented with at least two core clinical features of DLB (fluctuating cognition, visual hallucinations, parkinsonism, and/or rapid eye movement sleep behavior disorder) or one core clinical feature with at least one indicative biomarker. The indicative biomarkers included reduced dopamine transporter uptake in the basal ganglia demonstrated by single-photon emission computed tomography (SPECT) or positron emission tomography (PET), abnormal (low uptake) 123-Iodine-MIBG myocardial scintigraphy, and polysomnographic confirmation of RBD or a positive RBD screening questionnaire (RBD-SQ). (3) For patients enrolled prior to the publication of the 2017 criteria, diagnostic data were retrospectively re-evaluated against the current criteria using our databases to ensure consistency. (4) All enrolled patients showed relative preservation of medial temporal lobe structures on structural neuroimaging (MRI and/or CT). The clinical diagnosis of all patients was confirmed by a consensus agreement of at least two experienced neurologists following a comprehensive case review. Patients meeting any of the following exclusion criteria were excluded from this study: (1) Presence of severe neurological or psychiatric conditions that would impede compliance with study protocols, including severe visual/auditory impairment, aphasia, limb paralysis, or severe mental disorders, and/or inability to complete required clinical evaluations (such as neuropsychological assessments, neuroimaging examinations, polysomnography, or other procedures) due to the aforementioned conditions. (2) Lack of reliable caregivers to provide necessary clinical information or assist with study participation. (3) Patients with acute cardiovascular or cerebrovascular events (e.g., myocardial infarction or stroke). (4) Those diagnosed with neurodegenerative disorders potentially associated with dementia, including PD, Alzheimer’s disease, frontotemporal dementia, multiple system atrophy, progressive supranuclear palsy, or corticobasal degeneration. To ensure the study sample represented the broad clinical spectrum of DLB, no specific inclusion or exclusion criteria were applied based on dementia severity thresholds. This approach was adopted to enhance the generalizability of our findings regarding PS across different stages of the disease.

### 2.3 Enrollment and exclusion criteria for PS patients

Patients with PS exhibited trunk lateral flexion of at least 10° as measured by a wall-mounted goniometer. Those presenting with postural disorders (e.g., primary dystonia, scoliosis >10°, antecollis ≥45°), major spinal surgery, muscular and/or skeletal pathologies (e.g., myopathy, myositis, ankylosing spondylitis, rheumatoid arthritis), or inability to complete postural assessments were excluded.

### 2.4 Clinical data collection and neuropsychological assessment

This present study collected demographic data, medical history, clinical symptoms, past medical history, personal history, family history, and medication history. A comprehensive neuropsychological assessment was carried out by well-trained neuropsychologists face-to-face. Global cognition was evaluated using the Montreal Cognitive Assessment (MoCA), and visuospatial abilities were specifically tested with the Clock Drawing Test (CDT). Depressive and neuropsychiatric symptoms were measured using the Hamilton Depression Rating Scale (HAMD) and the Neuropsychiatric Inventory (NPI), respectively. Dementia severity was staged using the Clinical Dementia Rating (CDR) Scale. Daily living functioning was evaluated employing the Activities of Daily Living (ADL) Scale. Finally, caregiver burden was assessed using the Zarit Burden Interview (ZBI).

### 2.5 Neuropsychological tools

The MoCA (Nasreddine et al., 2005) is a clinician-administered, 30-point test that provides a global assessment of cognitive function. It assesses multiple domains, including visuospatial/executive abilities, naming, attention, language, abstraction, delayed recall, and orientation. Score range: 0 to 30. Interpretation: Lower total scores indicate more severe cognitive impairment. The CDT (Hazan et al., 2018) is a clinician-administered test that specifically evaluates visuospatial constructional ability and executive function. Score range: 0 to 5. Interpretation: A lower score indicates greater impairment. The total MoCA score, its specific subdomain scores, and the CDT score were used in the analysis.

The NPI (Cummings et al., 1994) is a structured, informant-based interview conducted with the patient’s caregiver. It assesses the presence, frequency, and severity of 12 neuropsychiatric symptoms (e.g., delusions, hallucinations, agitation, depression, anxiety, and aberrant motor behavior). Score calculation: A composite score (frequency × severity) is calculated for each symptom (range 0–12 per symptom) and summed to create a total score. Total score range: 0 to 144. Interpretation: Higher scores indicate more severe neuropsychiatric disturbances. The total NPI score, as well as the frequency, severity, and composite scores for individual symptoms was used in the analysis. The HAMD (Hamilton, 1967) is a clinician-administered scale used to quantify the severity of depressive symptoms in patients. The 17-item version was used. Total score range: 0 to 52. Interpretation: A higher total score indicates more severe depression. Scores used in analysis: The total HAMD score.

The CDR (Morris, 1993) is a clinician-rated scale derived from semi-structured interviews with both the patient and a reliable informant. It stages dementia severity by assessing performance in six cognitive and functional domains: memory, orientation, judgment & problem solving, community affairs, home & hobbies, and personal care. Scoring: A global CDR score is algorithmically derived. Global score categories: 0 = no dementia, 0.5 = questionable dementia, 1 = mild dementia, 2 = moderate dementia, 3 = severe dementia. For comparative analysis, patients were categorized into two groups: MCI and mild (CDR 0.5–1) and moderate–severe (CDR 2–3) dementia.

The ADL scale (Roberts et al., 2017) is an informant-based report that assesses a patient’s functional independence in performing basic (e.g., feeding, dressing) and instrumental (e.g., managing finances, using transportation) activities of daily living. Interpretation: The total score is used, with higher scores indicating greater functional impairment and dependency.

The ZBI (Zarit et al., 1980) is a self-report questionnaire completed by the primary caregiver to measure the subjective burden associated with caregiving responsibilities. Interpretation: Higher total scores indicate a greater perceived burden and psychological distress. Scores used in analysis: The total ZBI score.

### 2.6 Statistical analyses

Continuous variables were expressed as mean ± standard deviation (SD) when normally distributed or as median (interquartile range) for non-normally distributed data. Normality was assessed using the Shapiro–Wilk test. Between-group comparisons were performed using Student’s *t*-test for parametric data and the Mann–Whitney *U*-test for nonparametric data. Categorical variables were summarized as frequencies (*n*) with percentages (%) and analyzed using *χ*^2^ test or Fisher’s exact test as appropriate. Ordinal data were presented as median (quartiles) and analyzed with the Mann–Whitney *U*-test. All statistical analyses and data management were performed using SPSS 26.0 for Mac (IBM Corporation, Armonk, NY, United States). Graphical presentations were created using GraphPad Prism 9 software (GraphPad Software, San Diego, CA, United States) following established scientific visualization standards. All *p*-values reported are two-tailed, and *p* < 0.05 was considered statistically significant.

## 3 Results

The demographic and clinical features of DLB patients with and without PS are summarized in Table 1. Among the 218 DLB patients, 35 patients (19 males, 54.3%) presented with PS at the time of evaluation. Patients with PS had a significantly longer disease duration (56.46 ± 35.68 vs. 69.31 ± 33.29, *p* = 0.016) and presented with higher scores of ADL (39.60 ± 16.20 vs. 48.06 ± 15.54, *p* = 0.002) and prevalent of familial history of dementia (40.0% vs. 20.8%, *p* = 0.014). No significant difference was found for sex, mean age of onset, education level, marriage, caregiver, smoking, alcohol consumption, diabetes mellitus, hypertension, heart disease, stroke, and HAMD scores between the two groups.

**Table 1:** Demographic and clinical features of DLB patients with and without PS.

Characteristics	DLB without PS (*n* = 183)	DLB with PS (*n* = 35)	*p*-value
Sex (male *n*, %)	86 (47.0%)	19 (54.3%)	0.429
Mean age of onset (years)	68.29 ± 7.68	68.17 ± 9.07	0.936
Disease duration (months)	56.46 ± 35.68	69.31 ± 33.29	<0.05
Education (years)	9.93 ± 4.32	9.09 ± 4.30	0.218
Marriage (*n*, %)			0.961
Married	151 (82.5%)	29 (82.9%)	
Divorced and widow	32 (17.5%)	6 (17.1%)	
Caregiver (*n*, %)			0.656
Spouse	141 (77.0%)	26 (74.3%)	
Children	37 (20.2%)	7 (20.0%)	
Nursing professional	5 (2.7%)	2 (5.7%)	
Smoking, yes (*n*, %)	56 (30.6%)	12 (34.3%)	0.666
Alcohol consumption, yes (*n*, %)	38 (20.8%)	9 (25.7%)	0.514
Diabetes mellitus, yes (*n*, %)	28 (15.3%)	5 (14.3%)	0.878
Hypertension, yes (*n*, %)	75 (41.0%)	17 (48.6%)	0.405
Heart disease, yes (*n*, %)	33 (18.0%)	6 (17.1%)	0.900
Stroke, yes (*n*, %)	23 (12.6%)	6 (17.1%)	0.465
Familial history of dementia, yes (*n*, %)	38 (20.8%)	14 (40.0%)	<0.05
CDR (*n*, %)			<0.05
MCI and mild	69 (37.7%)	6 (17.1%)	
Moderate and severe	114 (62.3%)	29 (82.9%)	
HAMD	6.45 ± 6.51	8.34 ± 6.38	0.061
ADL	39.60 ± 16.20	48.06 ± 15.54	<0.01

DLB, dementia with Lewy bodies; PS, Pisa syndrome; CDR, Clinical Dementia Rating; MCI, Mild Cognitive Impairment; HAMD, Hamilton Depression Scale; ADL, Activities of Daily Living; *p* < 0.05 significant difference.

**Figure 1:**
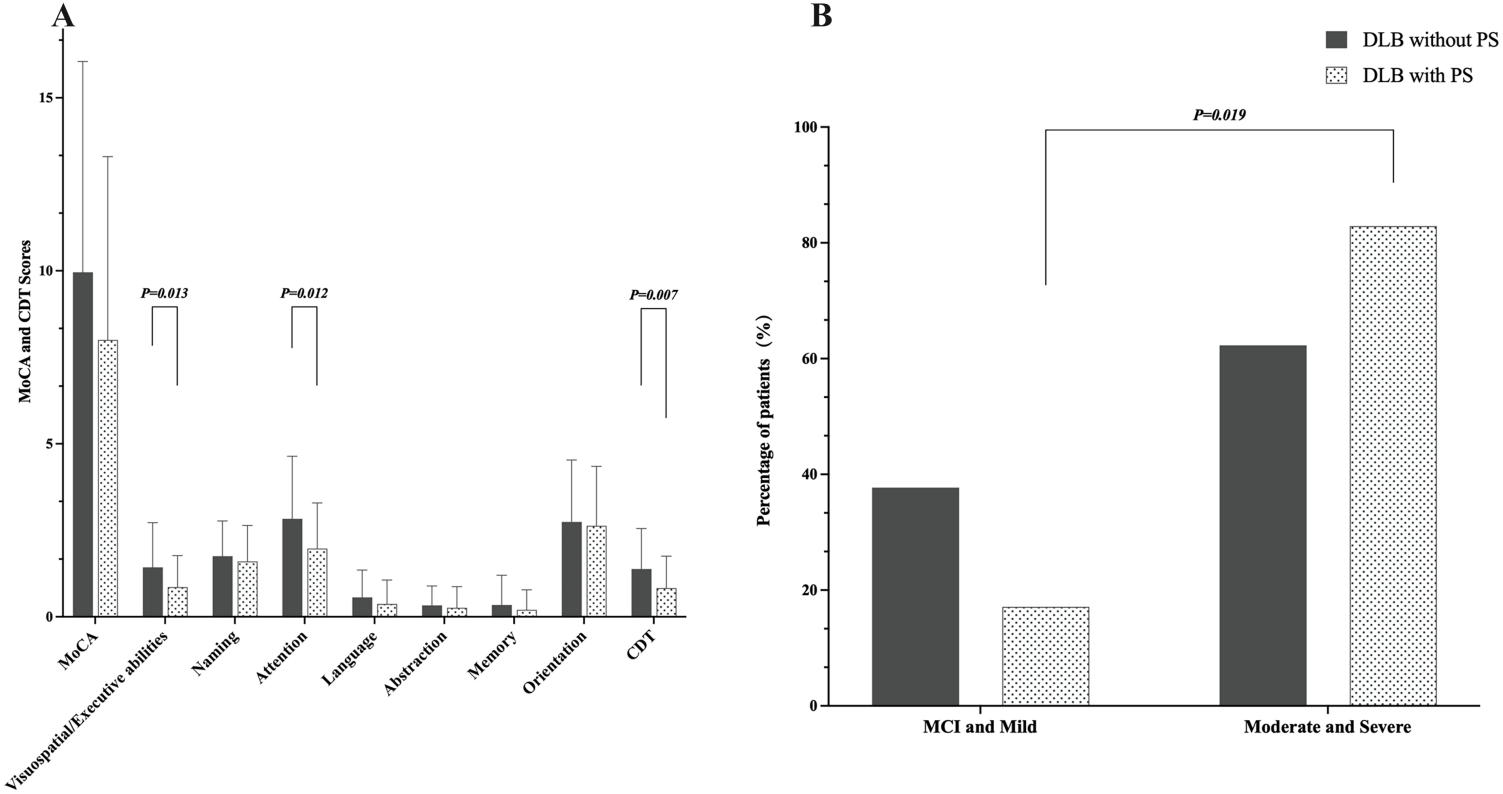
Cognitive profiles of DLB patients with and without PS. **(A)** Scores across cognitive domains assessed by MoCA and CDT. Data are presented as mean ± standard deviation in each group (*n* = 35 for DLB with PS; *n* = 183 for DLB without PS). Between-group comparisons were performed using the Mann–Whitney U test for all domains. **(B)** Distribution of dementia severity based on CDR scale. Data are presented as the percentage of patients. Between-group comparison was analyzed using the Chi-square test. DLB, dementia with Lewy bodies; PS, Pisa syndrome; CDR, Clinical Dementia Rating; MCI, Mild Cognitive Impairment; MoCA, Montreal Cognitive Assessment; CDT, Clock Drawing Test. *p* < 0.05 was considered statistically significant.

Comparisons of cognitive assessment results between DLB patients with and without PS are presented in Figure 1. The global cognitive evaluation did not show a significant difference between the two groups, with a MoCA score of 8.00 ± 5.30 for PS + vs. 9.96 ± 6.09 for patients without PS (*p* = 0.113). However, the PS + group showed a significantly worse performance in visuospatial/executive abilities (1.43 ± 1.29 vs. 0.86 ± 0.91, *p* = 0.013) and attention (2.83 ± 1.81 vs. 1.97 ± 1.32, *p* = 0.012) in the subscores of MoCA. As well, patients with PS presented with lower scores of CDT (1.38 ± 1.17 vs. 0.83 ± 0.92, *p* = 0.007). No significant differences were observed in the other cognitive domains evaluated. In addition, a higher proportion of patients with PS were in the moderate and severe dementia groups than in the mild and MCI groups, compared to individuals without PS (82.9% vs. 17.1%, *p* = 0.019).

**Figure 2:**
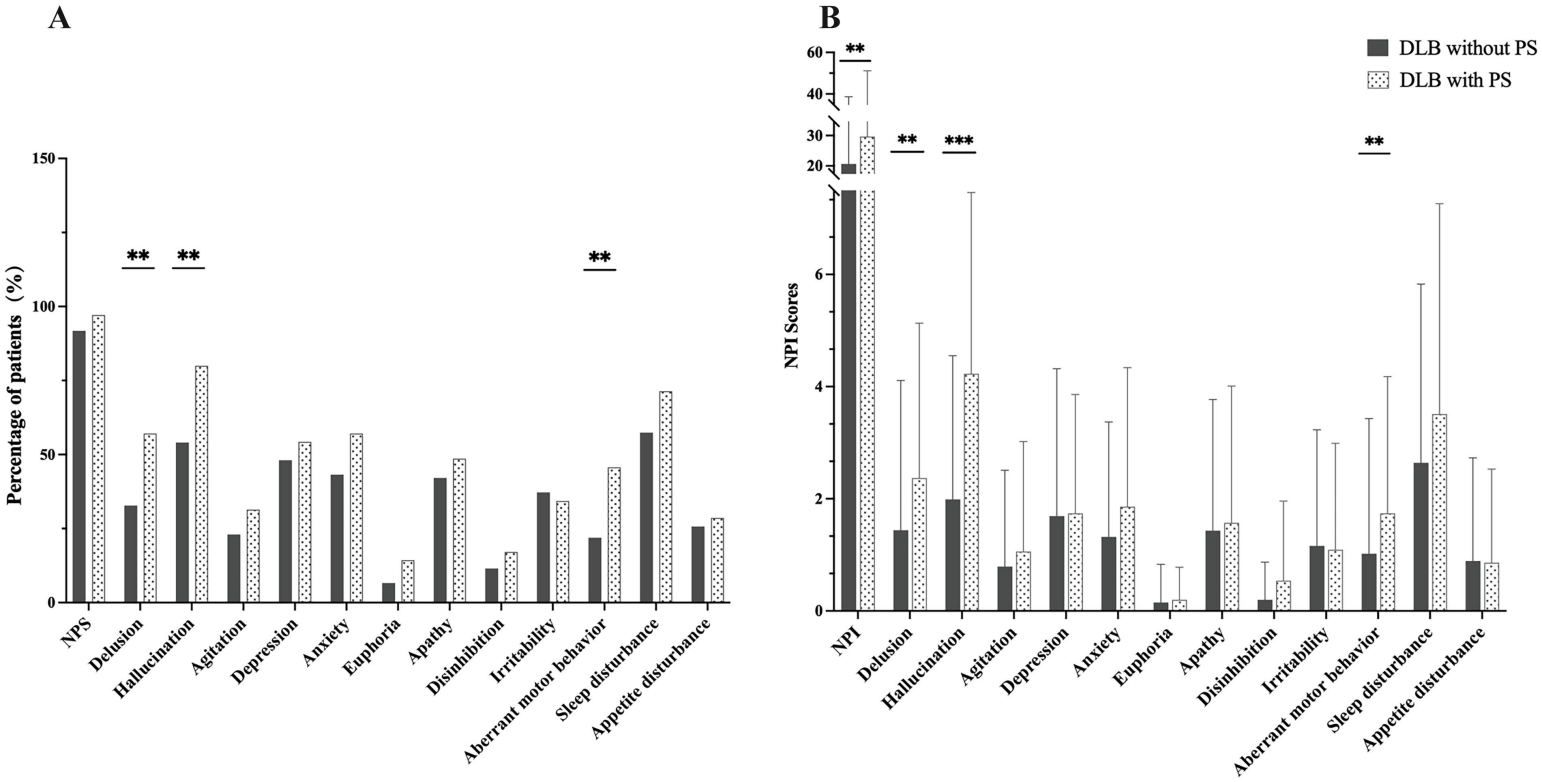
Neuropsychiatric symptoms in DLB patients with and without PS. **(A)** Prevalence of specific neuropsychiatric symptoms, as measured by the NPI. Data are presented as the percentage of patients in each group (*n* = 35 for DLB with PS; *n* = 183 for DLB without PS). Between-group comparisons for each symptom were analyzed using the Chi-square test. **(B)** Severity of neuropsychiatric symptoms, represented by the composite score from the NPI. Data are presented as mean ± standard deviation. Between-group comparisons were performed using the Mann–Whitney *U*-test. DLB, dementia with Lewy bodies; PS, Pisa syndrome; NPI, Neuropsychiatric Inventory; NPS, neuropsychiatric symptoms; *p* < 0.05 was considered statistically significant. ^∗∗^*p* < 0.01; ^∗∗∗^*p* < 0.001.

Figure 2 shows the incidence and scores of neuropsychiatric symptoms between DLB patients with and without PS. Comparative analysis revealed that DLB patients with PS had a significantly higher prevalence and severity of specific neuropsychiatric symptoms. Notably, they exhibited a greater frequency of delusions (57.1% vs. 32.8%; *p* = 0.006), hallucinations (80.0% vs. 54.1%; *p* = 0.004), and aberrant motor behaviors (45.7% vs. 21.9%; *p* = 0.020) compared to patients without PS. Concordantly, the NPI total score was significantly elevated in the PS group (29.69 ± 21.50 vs. 20.66 ± 17.97; *p* = 0.008), with notably higher composite scores for delusions (2.37 ± 2.76 vs. 1.44 ± 2.67; *p* = 0.008), hallucinations (4.23 ± 3.23 vs. 1.99 ± 2.56; *p* < 0.001), and aberrant motor behaviors (1.74 ± 2.44 vs. 1.02 ± 2.41; *p* = 0.006). No significant differences were observed in the other neuropsychiatric manifestations evaluated.

Comparison of caregiver distress score between DLB patients with and without PS are showed in Table 2. The PS group demonstrated significantly higher scores on the ZBI score compared to the group without PS (26.52 ± 17.74 vs. 18.04 ± 18.69, *p* = 0.023). Caregivers of patients with DLB complicated by PS exhibited significantly greater distress severity compared to those caring for patients without PS (*p* = 0.011). Notably, within neuropsychiatric symptoms subdomains, caregivers in the DLB + PS group presented heightened care distress associated with delusion (*p* = 0.030), hallucination (*p* < 0.001), anxiety (*p* = 0.020), aberrant motor behavior (*p* = 0.001), and sleep disturbance (*p* = 0.009) when contrasted with the group without PS.

**Table 2:** Comparison of caregiver distress and burden between DLB patients with and without PS.

Variable	DLB without PS (*n* = 183)	DLB with PS (*n* = 35)	*p*-value
ZBI			
Total score^†^	18.04 ± 18.69	26.52 ± 17.74	<0.05
NPI caregiver distress			
Total score^‡^	4 (1,9)	7 (3,14)	<0.05
Delusion^‡^	0 (0,1)	1 (0,2)	<0.05
Hallucination^‡^	0 (0,1)	2 (0,3)	<0.001
Agitation^‡^	0 (0,0)	0 (0,1)	0.400
Depression^‡^	0 (0,1)	0 (0,1)	0.781
Anxiety^‡^	0 (0,1)	1 (0,2)	<0.05
Euphoria^‡^	0 (0,0)	0 (0,0)	0.365
Apathy^‡^	0 (0,1)	0 (0,1)	0.891
Disinhibition^‡^	0 (0,0)	0 (0,0)	0.084
Irritability^‡^	0 (0,1)	0 (0,1)	0.815
Aberrant motor behavior^‡^	0 (0,0)	0 (0,1)	<0.01
Sleep disturbance^‡^	0 (0,1)	1 (0,2)	<0.01
Appetite disturbance^‡^	0 (0,0)	0 (0,0)	0.735

DLB, dementia with Lewy bodies; PS, Pisa syndrome; ZBI, Zarit Burden Interview; NPI, Neuropsychiatric Inventory; IQR, interquartile range. Data are presented as ^†^ mean ± standard deviation (analyzed by Student’s *t*-test) or ^‡^ median (IQR) (analyzed by Mann–Whitney *U*-test). *p* < 0.05 was considered statistically significant.

## 4 Discussion

This study represents the first investigation into the correlations between PS and cognitive deficits, neuropsychiatric manifestations, and caregiver burden in patients with DLB. The findings demonstrate that DLB patients with PS exhibit poorer performance in specific cognitive domains (e.g., attention, visuospatial and executive functions) and higher incidence/severity scores of certain NPS (e.g., delusions, hallucinations, aberrant motor behavior). Furthermore, delusions, hallucinations, aberrant motor behavior, anxiety, and sleep disturbances in patients with PS impose greater distress on caregivers and are associated with an overall increased caregiver burden.

Postural control is maintained through the integration of motor, sensory, and cognitive functions (Borel and Alescio-Lautier, 2014). Postural control deficits are linked to cognitive impairment in PD patients (Amboni et al., 2013). Abnormal subjective visual perception in PD patients with PS may contribute to postural deformities (Di Matteo et al., 2011). Functional deficits in vertical perception in PS patients are associated with impaired integration of visual, vestibular, and somatosensory information (Scocco et al., 2014; Huh et al., 2018), which is not only a hallmark of PS but also a risk factor for its development (Huh et al., 2018).

Given the shared neuropathological features and the overlap in motor and cognitive profiles between DLB and PD, it is plausible that similar mechanisms underpin postural control deficits in both conditions (Vitale et al., 2016; Gonzalez et al., 2023). Retrospective studies (Liu et al., 2019a, b) have identified significant executive dysfunction in PD patients with PS. In advanced PD stages, PS correlates with impairments in attention, language, and visuoperceptual functions (Vitale et al., 2016; Artusi et al., 2019). Vitale et al. (2016) revealed a significant association between PS and altered attention and visuoperceptual functions in PD patients, suggesting that PS pathogenesis may involve abnormalities in the frontostriatal system and posterior cortical functions. Artusi et al. (2022) similarly demonstrated that poorer visuospatial performance and vestibular tone imbalance are significantly correlated with PS.

These findings in PD are particularly relevant to DLB, which is characterized by more pronounced and earlier deficits in attention, executive function, and visuospatial abilities compared to PDD (McKeith et al., 2017; Smirnov et al., 2020). The poorer visuospatial/executive performance and attention deficits observed in patients with PS support the potential role of visuospatial and attentional impairments in PS pathogenesis, possibly mediated by distorted perception of trunk positioning. The severity of trunk misperception in PS patients correlates with the degree of visuospatial deficits (Artusi et al., 2019; Artusi et al., 2023). Additionally, the inferior language performance in PS patients, particularly in processing nouns with perceptual/sensory content, may relate to posterior temporal lobe dysfunction (Palermo et al., 2021). Although our study did not identify significant language deficits in DLB patients with PS—possibly due to the limitations of the global cognitive assessment used—the findings of Artusi et al. (2019) in PD patients with PS suggest that language dysfunction, potentially related to posterior temporal lobe dysfunction, could be a domain worthy of future investigation in DLB populations with this postural syndrome. A higher proportion of moderate-to-severe dementia in patients with PS suggests that progressive cognitive decline may impair awareness of postural abnormalities, exacerbating PS severity.

Our study highlights that DLB patients with PS exhibit more severe NPS and greater impairment in activities of daily living, as evidenced by their significantly higher ADL scores. QoL in DLB is influenced by multiple factors (Roberts et al., 2017), including progressive cognitive decline that disrupts social/occupational functioning and ADL (Mirza et al., 2022; Guo et al., 2023). Ye et al. (2017) reported significantly reduced QoL in PD with PS patients, though some studies (Tinazzi et al., 2015) argue that PS itself may not be the primary determinant, with disease duration and other factors playing contributory roles. NPS in DLB are strongly associated with QoL deterioration (Mirza et al., 2022; Guo et al., 2023; Kang et al., 2023). Patients with PS typically have longer disease duration, more severe dementia, and more pronounced motor impairments, all of which may amplify neuropsychiatric manifestations and QoL decline.

Differences in NPS exist between PD patients with and without PS. Tinazzi et al. (2016) elucidated the clinical and pathophysiological mechanisms by which NPS may influence PS development in PD. Al-Wardat et al. (2023) proposed that chronic musculoskeletal pain in PD patients with PS, varying in intensity, differentially impacts NPS. Asymmetric basal ganglia output, abnormal proprioceptive integration, antipsychotic medication use, and cognitive dysfunction may collectively modulate the incidence and severity of NPS in PD patients (Barone et al., 2016; Tinazzi et al., 2016). Although direct evidence in DLB is scarce, the high prevalence and severity of NPS in DLB, coupled with shared pathophysiological substrates, suggest that the interplay between NPS and postural control observed in PD is highly likely to be present and potentially even more pronounced in DLB. Our findings of significantly more severe delusions, hallucinations, and aberrant motor behavior in DLB patients with PS provide direct clinical evidence for this intricate relationship.

DLB patients demonstrate higher NPS prevalence than other dementias (Aarsland, 2020; Schwertner et al., 2022), with these symptoms serving as both prodromal markers and risk factors for disease progression (Hamilton et al., 2021; Guo et al., 2023; Kang et al., 2023). Vivid visual and auditory hallucinations in DLB are linked to atrophy in parieto-occipital regions (McKeith et al., 2017), while delusions, anxiety, aberrant motor behavior, and sleep disturbance are also common (Elefante et al., 2021). Pennington et al.’s comparison of cognitive and NPS profiles between PD and DLB patients provides insights into NPS differences between patients with and without PS (Pennington et al., 2021). The concordance between higher incidence/severity of delusions, hallucinations, and aberrant motor behavior in patients with PS suggests that frequent and severe NPS exacerbates patient and caregiver distress, amplifying caregiver burden.

The frequency of NPS and the severity of psychiatric manifestations in patients with DLB may be closely associated with the degree of dementia or serve as early warning signs of disease progression (Kawada, 2019; Kim et al., 2021). These symptoms significantly impair caregivers’ quality of life, sleep, and mental health, exacerbating caregiver distress and caregiving burden (Kang and Hur, 2021; Rigby et al., 2021). NPS such as anxiety, apathy, depression, and cognitive decline or dementia are primary contributors to caregiver burden in PD patients (Rigby et al., 2021). Al-Wardat et al. (2023) and Tinazzi et al. (2016) demonstrated that axial postural abnormalities and dorsal pain in PD patients can exacerbate preexisting NPS in those with PS, rendering these symptoms more challenging to manage.

In our study, patients with PS exhibited more severe caregiver distress and a higher overall caregiving burden, particularly due to symptoms such as delusion, hallucinations, anxiety, aberrant motor behavior, and sleep disturbance. These findings suggest that PS and NPS synergistically interact, compounding caregiver distress and burden, while profoundly impacting caregivers’ QoL and mental health. Such comorbidities are highly prevalent among individuals with PD (Elefante et al., 2021). For caregivers of DLB patients with PS, who often contend with a double burden of severe NPS and disabling postural disorder, the heightened distress levels we observed underscore an urgent need for targeted support. This may provide critical insights for refining caregiving strategies and priorities, ultimately alleviating burden and improving the overall caregiving experience.

Our findings highlight the necessity for an integrated management strategy in DLB patients with PS. Clinically, the specific deficits in attention and visuospatial function call for tailored cognitive rehabilitation, while the severe neuropsychiatric burden requires careful symptom management and proactive caregiver support to alleviate the documented increase in distress and burden. Future research should prioritize longitudinal studies to establish causality, investigate the underlying neurobiological mechanisms, and develop targeted interventions aimed at mitigating PS progression and improving patient and caregiver outcomes.

This study has several limitations that should be acknowledged. Firstly, the findings are derived from a cross-sectional design, we are unable to determine whether such cognitive profile and neuropsychological changes are causative or consequential to the postural abnormality. Additionally, as no specific inclusion or exclusion criteria were applied based on dementia severity, the patient group was consequently relatively heterogeneous in terms of disease stage, which may confound the interpretation of our findings and hinder their generalizability. Secondly, although this is a multicenter study of DLB combined with PS, all participants were recruited from dementia clinics, potentially introducing selection bias. Therefore, our results require further validation through studies with larger sample sizes and multiple follow-up assessments. Finally, when we evaluate the impact of cognitive function and neuropsychological factors, we need to further apply more specific scales to re-evaluate and verify. For example, by Benton Judgment of Line Orientation (BJLOT) for visuo-spatial abilities, by Semantic Verbal Fluency (SVF) and Phonemic Verbal Fluency (PVF) for language, by Digit Cancellation Test (DCT) and Trail Making Test A (TMT A) for attention, etc.

## 5 Conclusion

In conclusion, our study delineates a distinct clinical phenotype associated with PS in patients with DLB. This phenotype is distinguished by severe impairments in attention and visuospatial/executive functions, coupled with a heightened severity of NPS, most notably delusions, hallucinations, and aberrant motor behavior. These specific cognitive profiles call for targeted rehabilitation strategies, while the pronounced neuropsychiatric burden necessitates integrated and carefully calibrated management in clinical implications. Most importantly, the significantly elevated caregiver burden underscores the critical need for robust support systems. Addressing this complex comorbidity through a multifaceted approach is paramount to improving patient outcomes, enhancing quality of life, and alleviating the profound distress experienced by caregivers.

## Data Availability

The raw data supporting the conclusions of this article will be made available by the authors, without undue reservation.
